# Clinical outcomes of COVID-19 infection in congenital heart disease: A single-center experience in Indonesia

**DOI:** 10.3389/fcvm.2022.1022183

**Published:** 2022-11-01

**Authors:** Sisca Natalia Siagian, Susandy Oetama, Fathy Zuandi Pohan, Brian Mendel, Olfi Lelya, Damba Dwisepto Aulia Sakti, Yovi Kurniawati

**Affiliations:** ^1^Division of Pediatric Cardiology and Congenital Heart Disease, Department of Cardiology and Vascular Medicine, National Cardiovascular Centre Harapan Kita, Universitas Indonesia, Jakarta, Indonesia; ^2^Division of Pediatric Cardiac Intensive Care, National Cardiovascular Centre Harapan Kita, Universitas Indonesia, Jakarta, Indonesia; ^3^Sultan Sulaiman Government Hospital, Serdang Bedagai, Indonesia

**Keywords:** clinical outcome, congenital heart disease, CHD (congenital heart disease), COVID-19, comorbidities

## Abstract

**Background:**

Congenital heart disease (CHD) patients are thought to be vulnerable to COVID-19 complications. In this study, we would like to assess the outcomes and clinical characteristics in COVID-19 CHD patients.

**Method:**

A single-center, observational study was conducted in National Cardiovascular Center Harapan Kita (NCCHK). This study included patients with CHD who were hospitalized for COVID-19. The extracted data were baseline characteristics, clinical findings, supportive examination findings, complications, outcomes, and length of stay of the patients. The data were then analyzed using SPSS 26.0 software.

**Result:**

Twenty-six patients with CHD and COVID-19 infection were included in our study. There were 24 resolved cases and 2 deaths, four patients experienced complications such as renal insufficiency (1), sepsis (2), and multiorgan failure (1). The median length of stay was 13 days. The most common symptoms experienced by the patients were breathlessness (65.4%), cough (57.7%), and fever (42.3%).

**Conclusion:**

We observed a relatively mild COVID-19 clinical course despite prior research showing that patients with cardiovascular comorbidities, such as CHD, have a higher case-fatality rate. This could be because of the smaller sample size, non-standardized diagnosis, severity, treatment, and age group.

## Introduction

Less than 5% of COVID-19 infections that have been diagnosed globally have been in children (1). One percent of COVID-19 instances were in children under the age of 10, according to a survey of 72,314 cases by the Chinese Center for Disease Control and Prevention (2–4) Due to COVID-19's negative impact on a pressure- or volume-overloaded heart in congenital heart disease (CHD), patients with CHD are thought to be at high risk for its negative effects (5).

Studies and knowledge about CHD and COVID-19 in children and adolescents are still unclear. Additionally, factors that affect how severe COVID-19 is in individuals with CHD are still unknown (6). Therefore, we carried out a study to evaluate the clinical traits and outcomes in CHD patients infected with COVID-19.

## Methods

### Study design and settings

Twenty-six CHD patients with COVID-19 were included in this prospective, single-center observational trial between January 2019 and February 2022 were admitted to cardiology services and few entered the intensive care unit (ICU). This study was conducted at the National Cardiovascular Center Harapan Kita (NCCHK) Jakarta, Indonesia. Institutional Review Board of National Cardiovascular Center Harapan Kita No. UM.01.05/2.2.2/175/2022 has ethically authorized this study. Patients included in this study were all hospitalized CHD patients for COVID-19 infection. The diagnosis of COVID-19 was defined as either “clinically suspected” or “confirmed”, where SARS-CoV-2 test had been performed and was positive. All patients receiving emergency, outpatient, and inpatient services are recorded in an electronic medical record (EMR) system that is kept up to date by the hospital. Patients who met the inclusion criteria were then followed-up and evaluated during the hospital stay.

### Follow-up and outcomes

The baseline characteristics of the subjects were extracted, including age, gender, systolic and diastolic pressure, heart rate, respiratory rate, oxygen saturation, and temperature. CHD classification was also described, followed by complication, resolution of COVID-19 or mortality, and length of stay. The patients were followed-up until they are discharged from the hospital. The clinical findings were recorded during hospitalization, including the severity of COVID-19 infection and the findings from chest X-rays, laboratory, and echocardiography examinations.

### Statistical analysis

The nominal data were presented as frequency and percentage. The Shapiro-Wilk test was used to assess the normality test for numerical data, where normally distributed data were represented by the mean and standard deviation or by the median and range if the data were skewed. The statistical analysis was carried out using Statistical Package for Social Science (SPSS) 26.0 software.

## Results

This study included 26 patients with CHD and COVID-19 infection. The baseline characteristics of the patients are summarized in Table 1. The patients include 15 females and 11 males with a median age of 4 (0–41). The mean systolic and diastolic pressures were 105.3 ± 62.7 mmHg and 21.4 ± 20.5 mmHg, respectively. The mean heart rate was 117.3 ± 30,5 beats per min, while the mean oxygen saturation was 77.7 ± 17 %. The median respiratory rate was 28 (16–46) breathes per min. The median temperature was 36.9 (36–39)° C. The most predominant CHD type included was restricted pulmonary blood flow (PBF) cyanotic CHD (45.8%). Four patients experienced complications, including renal insufficiency (3.8%), sepsis (7,7%), and multiorgan failure (3,8%). The median length of stay was 13 days, and the outcomes were 24 resolved cases and two deaths.

**Table 1:** Baseline characteristics.

	***n***	**%**	**Mean**	**Standard deviation**	**Median**	**Min-max**
Age (years)					4	0-41
Systolic (mmHg)			105.3	62.7		
Diastolic (mmHg)			21.4	20.5		
Heart rate			117.3	30.5		
Respiratory rate					28	16-46
Temperature (Celsius)					36.9	36-39
Saturation (%)			77.7	17		
Male	11	42.3	
**CHD type**	
Acyanotic	9	37.5	
Cyanotic, unrestricted	4	16.7	
Cyanotic, restricted	11	45.8	
**Comorbidity**	
Older age (>18 years)	3	11.5	
Obesity	2	7.7	
**Complication**	
Renal insufficiency	1	3.8	
Sepsis	2	7.7	
Multiorgan failure	1	3.8	
**Outcome**	
Resolved	24	92.3	
Died	2	7.7	
Length of stay					13	Feb-74

CHD, congenital heart disease.

Clinical manifestation and supportive examination findings are shown in Table 2. Various degrees of COVID-19 infections are observed among these patients: mild (26.9%), moderate (26.9%), severe (34.6%), and critical (11.5%). Given the data, seven adolescent (26.9%) and 11 children (42.3%) showed moderate to critical symptoms. The most common symptoms are breathlessness (65.4%), cough (57.7%), fever (42.3%), rhinorrhea (26.9%), diarrhea (7.7%), nausea (3.8%), and anosmia (3.8%). Chest X-ray imagings are diverse, ranging from oligemia (57.7%), normal (50%), plethora (42.3%), infiltrates (30.8%), to congestion (19.2%). The laboratory findings were found to be various among these patients. The complete blood count results were as follows: the median hemoglobin and hematocrit were 13.7 (9.7–23.2) mg/dL and 40.4 (29.4–68.7)%, respectively, while the mean leukocyte and thrombocyte were 9,201 ± 3,410/μL and 258,920 □ 111,063/μL, respectively.

**Table 2:** Clinical manifestation and supportive examination findings.

	***N***	**%**	**Mean**	**Standard deviation**	**Median**	**Min-max**
**Clinical findings/features**	
Fever	11	42.3	
Cough	15	57.7	
Rhinorrhea	7	26.9	
Breathlessness	17	65.4	
Nausea/vomitus	1	3.8	
Diarrhea	2	7.7	
Anosmia	1	3.8	
Ageusia	0	0	
**Severity**	
Mild	7	26.9	
Moderate	7	26.9	
Severe	9	34.6	
Critical	3	11.5	
**Chest X-ray**	
Normal	13	50	
Infiltrate	8	30.8	
Congestion	5	19.2	
Oligemia	15	57.7	
Plethora	11	42.3	
**Laboratory findings**	
Hb					13.7	9.7-23.2
Ht					40.4	29.4-68.7
Leukocyte			9201	3410		
Thrombocyte			258920	111063		
Seg absolute			3974	1947		
Lym absolute			3964	2458		
NLR		1.22	0.18-8.69
ESR					6	Jan-88
CRP					2	< 1-76
D-dimer					700	190-87826
Fibrinogen			289	83.7		
Ureum					25.8	6.8-213.2
Creatinine					0.39	0.18-3.92
CT value					25.2	14.7-36.2
**Echocardiographic findings**	
EF (%)			71.4	9.2		
TAPSE (cm)					1.7	0.8-2.2

CRP, C-reactive protein; CT, cycle threshold; EF, ejection fraction; ESR, erythrocyte sedimentation rate; Hb, hemoglobin; Ht, hematocrit; NLR, neutrophil-lymphocyte ratio; TAPSE, tricuspid annular plane systolic excursion.

The mean absolute count of segmented neutrophils and lymphocytes was 3,974 □ 1,947 and 3,964 ± 2,458, respectively. The median neutrophil-lymphocyte ratio was 1.22 (0.18–8.69). The inflammation biomarkers assessed in this study were erythrocyte sedimentation rate (ESR) and C-reactive protein (CRP). The median ESR and CRP were 6 (1–88) mm/h and 2 (<1–76) mg/dL, respectively. The mean fibrinogen was 289 ± 83.7 mg/dL, while the median D-dimer was 700 (190–87,826) ng/mL, ureum and creatinine were 24.8 (6.8–213.2) mg/dL, and 0.39 (0.18–3.92) mg/dL, respectively. The median CT value was 25.2 (14.7–36.2). In echocardiographic findings, the mean ejection fraction was 71.4 ± 9.2%, while the median tricuspid annular plane systolic excursion (TAPSE) was 1.7 (1.8–2.2) cm. Table 3 shows the individual characteristics, medications, and outcomes of the included patients.

**Table 3:** The individual characteristics, medications, and outcomes of the included patients.

**#**	**Patient data**	**Diagnosis**	**CHD Type**	**Surgery**	**Symptoms**	**Weight (kg)**	**SpO2 (%)**	**CT value**	**Initial Leukocyte**	**NLR**	**D-dimer**	**Cr**	**CXR**	**EF (%)**	**TAPSE (mm)**	**Severity**	**Medication**	**Complication**	**ETT**	**LoS (days)**	**Clinical Outcome**
1	Girl, 15-year-old	SA, RA isomerism, MA, PA, ASD, moderate TR	Cyanotic, restricted pulmonary blood flow	Yes	Fever, cough, common cold, anosmia	25	45	31.3	8380	1.49	350	0.75	Infiltrate	-	13.5	Moderate	Antiviral, antibiotic, diuretic, ACEi, B, PDE5i	-	No	37	Resolved
2	Boy, 4-month-old	TA, hypoplastic RV, secundum ASD (R-L shunt), muscular VSD (L-R shunt), mild PS	Cyanotic, unrestricted pulmonary blood flow	No	Asymptomatic	5.5	91	-	12870	0.18	989	0.28	Infiltrate	71	-	Mild	Antibiotic, diuretic, MRA	-	No	2	Resolved
3	Boy, 17-year-old	Perimembranous VSD (L-R shunt)	Acyanotic	No	Fever, cough	30	98	-	6620	1.38	-	0.58	Normal	74	25	Moderate	Antibiotic	Endocarditis	No	2	Resolved
4	Girl, 1-year-old	Tetralogy of Fallot with cyanotic spell	Cyanotic, restricted pulmonary blood flow	No	Fever	8	51	-	11100	2.02	-	0.34	Normal	68	15	Mild	Antibiotic, BB	-	No	16	Resolved
5	Boy, 17-year-old	CTEPH, TR severe due to PH, AFib NVR	Miscellaneous	No	Breathlessness	45	89	21.8	15300	1.48	-	0.62	Congestion	65	9	Mild	Antiviral, antibiotic, diuretic, ACEi, BB, MRA, PDE5i	-	No	9	Resolved
6	Girl, 1-year-old	TAPVD, DORV, VSD SADC, SVD without PS, PDA, moderate TR due to PH	Cyanotic, unrestricted pulmonary blood flow	No	Cough, common cold	6	86	34	9940	0.88	-	0.23	Infiltrate	63	8	Mild	Antibiotic, diuretic, ACEi,	-	No	11	Resolved
7	Boy, 3-year-old	TA-inlet VSD, secundum ASD, severe PS valvar-infundibular, hypoplastic RV, PDA, MAPCAs	Cyanotic, restricted pulmonary blood flow	R-BT shunt and PDA ligation	Asymptomatic	11	80	33.2	5307	1.07	-	0.32	Normal	83	-	Mild	Antibiotic, ACEi, BB, anticoagulant	Sepsis	No	11	Resolved
8	Boy, 4-year-old	Tetralogy of Fallot	Cyanotic, restricted blood flow	Yes	Cough, breathlessness	5	63	23.7	7110	1.96	7532	0.46	Infiltrate	60	16	Critical	Antiviral, antibiotic, diuretic, ACEi, steroid, anticoagulant	-	Yes	32	Resolved
9	Boy, 9-month-old	Intracardiac TAPVD	Cyanotic, unrestricted pulmonary blood flow	No	Breathlessness	4	85	27.1	12070	0.26	597	0.38	Normal	76	10	Moderate	Antiviral, antibiotic, diuretic, ACEi	-	No	27	Resolved
10	Boy, 11-old-month	PA-subaortic VSD, overriding aorta, mild MS, suspect bilateral SVC, stenotic PA bifurcatio	Cyanotic, restricted pulmonary blood flow	No	Breathlessness	7	60	25.1	7470	0.41	322	0.36	Infiltrate	66	16	Mild	Antiviral, antibiotic, BB	-	No	12	Resolved
11	Boy, 4-year-old	Tetralogy of Fallot	Cyanotic, restricted pulmonary blood flow	Yes	Breathlessness	12	67	14.7	16460	0.72	3308	0.36	Normal	73	11	Moderate	Antibiotic	-	No	18	Resolved
12	Girl, 7-month-old	PA-CAVSD, PDA L-R shunt, mesocardia, SA, LA isomerism, moderate AV valve regurgitation, LV smallish	Cyanotic, restricted pulmonary blood flow	No	Fever, cough, breathlessness	4	60	34.5	7660	1.22	1033	0.31	Normal	-	8	Severe	Antiviral, antibiotic, diuretic, ACEi, MRA, anticoagulant		Yes	28	Resolved
13	Girl, 17-year-old	RHD, right HF, severe MR, severe TR	Miscellaneous	No	Fever, cough, common cold, breathlessness	47	98	14.9	8630	1.79	4181	0.53	Congestion	62	16	Severe	Antiviral, antibiotic, diuretic, ACEi, BB, steroid, anticoagulant	-	No	29	Resolved
14	Girl, 17-year-old	Perimembranous VSD L-R shunt, possible IE	Acyanotic	No	Cough	35	97	34.6	6370	2.42	423	0.59	Normal	71	20	Mild	Antibiotic, diuretic, ACEi, BB	Endocarditis	No	7	Resolved
15	Girl, 7-month-old	TA, PA-IVS post PDA stenting and BAS	Cyanotic, restricted pulmonary blood flow	No	Fever, breathlessness	5	69	15.8	6760	0.34	1534	0.52	Normal	78	-	Critical	Antiviral, antibiotic, diuretic, MRA, steroid, anticoagulant	Sepsis	Yes	20	Died
16	Boy, 7-month-old	ASD secundum, mild PS valvar	Acyanotic	No	Fever, cough, common cold, diarrhoea, breathlessness	4.1	41	16.8	15440	0.85	87826	0.56	Normal	91	1.7	Severe	Antibiotic, diuretic, ACEi, anticoagulant	-	No	19	Resolved
17	Girl, 11-year-old	Severe PS valvar, moderate PR, PDA, post PVR bioprosthetic	Acyanotic	Yes	Fever, cough, breathlessness	25	90	21.2	10940	2.35	642	0.4	Normal	80	15	Moderate	Antiviral, antibiotic, diuretic, ACEi, BB, anticoagulant	Endocarditis	Yes	74	Resolved
18	Boy, 6-year-old	SI, ventricular inversion, MA, DOLV-CAVSD, severe PS	Cyanotic, restricted pulmonary blood flow	No	Fever, cough, breathlessness	13	74	33.1	10100	0.54	4523	0.33	Infiltrate	58	-	Severe	Antiviral, antibiotic, BB, anticoagulant	-	No	29	Resolved
19	Girl, 10-month-old	Perimembranous VSD	Acyanotic	No	Fever, cough, diarrhoea, breathlessness	6	85	26.4	14910	0.48	430	0.27	Infiltrate	-	-	Severe	Antiviral, antibiotic, diuretic, ACEi, BB, MRA, steroid	-	No	13	Resolved
20	Girl, 8-month-old	Perimembranous VSD	Acyanotic	No	Fever, cough, diarrhoea, breathlessness	5	99	18.9	8140	0.22	620	0.28	Congestion	68	13	Severe	Antiviral, antibiotic, diuretic, ACEi	-	No	7	Resolved
21	Girl, 15-year-old	LA isomerism, partial AVSD, large PDA, bilateral SVCs, moderate MR due to cleft, post PA banding and PDA ligation	Cyanotic, unrestricted pulmonary blood flow	Yes	Cough	45	73	32.6	6770	8.69	190	0.56	Congestion	79	-	Severe	Antiviral, diuretic, ACEi, MRA, steroid, anticoagulant	-	No	5	Resolved
22	Girl, 8-year-old	Tetralogy of Fallot, CAVSD, post RVOT stenting (April 2021)	Cyanotic, restricted pulmonary blood flow	No	Fever, cough, common cold	17	85	25.2	2740	1.71	1730	2.87	Normal	67	26	Moderate	Antiviral, antibiotic, diuretic, ACEi, BB	Acute kidney injury	No	11	Resolved
23	Girl, 11-month-old	PDA	Acyanotic	No	Fever, cough, common cold, diarrhoea, breathlessness	6	84	15.2	12090	1.73	700	0.18	Congestion	78	13	Severe	Antiviral, antibiotic, diuretic, ACEi, BB	-	No	10	Resolved
24	Male, 26-year-old	PDA L-R shunt, mild-moderate MR, mild TR	Acyanotic	No	Asymptomatic	64	100	-	5680	1.79	993	1.03	Normal	55	22	Moderate	Antiviral, antibiotic, diuretic, ACEi, BB, anticoagulant	Endocarditis, acute kidney injury	No	18	Resolved
25	Female, 27-year-old	TA, secundum ASD, hypoplastic RV, muscular VSD post BCPS	Cyanotic, restricted pulmonary blood flow	Yes	Breathlessness	33	67	33.1	6100	1.02	300	0.74	Normal	60	-	Severe	Antiviral, antibiotic, ACEi, anticoagulant	-	No	13	Resolved
26	Female, 41-year-old	Right HF due to primary PH, PFO, severe TR	Miscellaneous	No	Breathlessness	200	85	36.2	10130	8.33	-	3.92	Infiltrate	70	12	Critical	Antibiotic, diuretic, ACEi, MRA, PDE5i, anticoagulant	Acute kidney injury	No	3	Died

## Discussions

Clinicians need to be aware of the difficulties in diagnosing COVID-19 in children with untreated CHD. A subset of COVID-19-treated CHD patients had myocardial injury, as evidenced by changes in the electrocardiogram and increased levels of troponin, creatine kinase (CK), creatine kinase-MB (CK-MB), and the N-terminal fragment of brain natriuretic peptide (BNPP) (NT-Pro-BNP) in about 8-12% of patients (6–8). The presumed cause of death could be severely reduced ventricular function and acute heart failure. Viral myocarditis, hypoxia, stress cardiomyopathy, or systemic hyperinflammation could all be factors. Right ventricular failure caused by increased pulmonary capillary resistance and mechanical ventilation could be an ARDS complication. Transient, reversible left ventricular dysfunction, and cardiogenic shock have also been reported (9–12).

Arrhythmia has been reported in a significant number of critically ill COVID-19 patients, including cases of sudden cardiac death. Myocarditis, ventricular dysfunction, electrolyte imbalances, fever, and endogenous or exogenous inotropes can all increase the risk. Recent reports have also highlighted the occurrence of venous and arterial thrombosis, which may be related to immobility, hypoxia, hyperinflammation, and diffuse intravascular coagulation (7, 11–14).

From a total of 105 patients included in the study, Schwerzmann, et al. (15) found that those with a complicated illness course were older and more likely to be overweight than those with ACHD and uncomplicated COVID-19. The genders of patients with and without a challenging COVID-19 course did not differ significantly. Between patients with and without extensive COVID-19, there was no difference in the overall complexity of heart defects. Their work emphasizes the significance of a thorough risk assessment, which should encompass general risk factors and comorbidities for risk estimation in COVID-19 in addition to the underlying congenital abnormality. Our study also showed worse clinical outcomes in CHD patients with older age and obesity.

The ACHD population hardly ever contains acquired cardiovascular and other comorbidities linked to fatal COVID-19 results. In the Spanish ACHD cohort, 75% of patients were under the age of 45, and only 14, 2.7, and 1.5% of patients had ischemic heart disease, hypertension, or diabetes, respectively (15, 16).

### Specific aspects of congenital heart disease

Studies have shown that underlying cardiovascular diseases increase the morbidity and mortality rates in those infected with SARS-CoV-2. However, the virus itself has been shown to cause cardiovascular complications. Individuals with CHD are also susceptible not only to cardiovascular complications but also to non-cardiovascular complications (17–24). Based on Kordzadeh-Kermani E, et al. (25), complications of COVID-19 included multi-organ involvement and sepsis (24–27). A systematic review from Haiduc AA, et al. (28), stated that patients with more severe CHD were prone to COVID-19 complications due to their poor functional capacities. The severity of adult patients with CHD may also vary depending on the status of surgical repair, the anatomical complexity, and other physiological conditions (29). A cohort study from Italy reported that 9% of 76 patients with CHD and COVID-19 developed heart failure as the most common complication, followed by arrhythmias, stroke, and pulmonary hypertension (similar prevalence of 3%) (30). A survey from Simpson M, et al. (31), found that all seven pediatric patients with CHD and COVID-19 developed acute decompensation heart failure, with one death in an 18-year-old with hypertrophic cardiomyopathy. In a multicentered study, there were 24 COVID-19-related deaths from 1,044 infected adult patients with CHD. Diabetes, being a man, cyanosis, pulmonary hypertension, renal insufficiency, and previous heart failure hospitalization were all risk factors for death.

A structural CHD did not necessarily indicate an increased risk of mortality or morbidity in COVID-19. Out of 26 patients in our center, eight patients suffered from complications. We found three patients with endocarditis, two patients with sepsis, two patients with acute kidney injury, and one with both endocarditis and acute kidney injury. While endocarditis was previously reported as a complication of COVID-19 in adults and none in children, our study found three pediatric patients and one adult patient with endocarditis. It is yet unclear whether factors associated in the pathophysiology of COVID-19 infection can increase IE risk. Sentinel findings based on animal models of infection raised the possibility that a second independent mechanism may involve cardiac valve inflammation with expression of surface structures to enhance bacterial adhesion in addition to the possibility that damaged endothelium can predispose to IE caused by *S.aureus*. It is evident from earlier COVID-19-related experiments that endothelitis can have an effect on vascular endothelial surfaces (12). Two cyanotic pediatric patients developed sepsis during hospitalization, and one of them eventually died due to sepsis. Acute kidney injury was also found in an older patient with CHD. Two of the three adult patients with CHD in our study developed acute kidney injury, and one died during hospitalization. Alsaied T, et al. (22), stated that the risk of severe COVID-19 in a patient with CHD is unclear. It is reasonable that those with severe CHD are at higher risk of having COVID-19, followed by higher severity. Memar EHE, et al. (5) noted in a case series consisting of nine pediatric patients with COVID-19 and CHD, of which two patients presented with severe COVID-19 and died during hospitalization, that both patients had severe forms of CHDs: one with aortic stenosis and the other with hypoplastic left heart syndrome. In our study, two patients who died had severe forms of CHD. The patient with tricuspid atresia and pulmonary atresia with intact ventricular septum had undergone PDA stenting and balloon atrial septectomy with an episode of severe low cardiac output syndrome. The other patient had cardiogenic shock due to severe primary pulmonary hypertension with right heart failure. This finding is in line with the study by Lewis MJ, et al. (32), that reported pulmonary hypertension was one of the strongest predictors for moderate to severe COVID-19 in all patients with CHD. The severity of COVID-19 was assessed mainly based on the clinical features. Therefore, our findings on nearly 50% of subjects presenting severe and critical COVID-19 should be analyzed further. These findings might be due to the impact of CHD on COVID-19 progression, the worsening of the CHD condition itself, the interaction between both conditions, or possible bias caused by the study population of only hospitalized patients (33–35).

### Symptoms of congenital heart disease with COVID-19

Children were less likely to contract COVID-19, and the majority of infected kids had minimal to no symptoms (13–15). In this study, we found that breathlessness, cough, fever, rhinorrhea, and diarrhea as the five most common symptoms of COVID-19 in CHD patients regardless of their ages, and only 3 out of 26 were presenting with gastrointestinal symptoms. Anosmia and ageusia are challenging to be assessed in young children, therefore, only one patient was reported having anosmia. While a cohort of 37 children in the North region of Iran reported Multisystem Inflammatory Syndrome in children (MIS-c) as their primary manifestation in (18.9%) (17), this finding was not confirmed in our study. In a systematic analysis by Ahmed, et al. (19), 71% of 662 patients with MIS-c were admitted to the intensive care unit (ICU), with 1.7% mortality. The most common clinical manifestations were fever (100%), diarrhea or abdominal pain (73.7%), and vomit (68.3%), with more than half of the patients had left ventricular systolic dysfunction. A decrease in cardiac function was also found in the COVID-19 patients with comorbid CHD, shown by a relatively lower TAPSE found in all patients. In contrast, the echocardiography baseline data in our study have shown that all patients had good LV and RV function, with normal LV EF and TAPSE.

According to several research from China, 12–30% of pediatric COVID-19 patients had testing results that were positive, while the remaining patients had very suspicious clinical manifestations. Only 42% of patients were febrile, some individuals had no symptoms, and only 0.02% required critical care (13, 20). Different from our study, all CHD patients infected with COVID-19 showed symptoms varied from mild to severe and critical. In the course of admission, four patients required intensive care unit and mechanical ventilation, of which two died. Nonetheless, the proposed belief that pediatric patients with COVID-19 elicited milder symptoms than adults does not apply to CHD cases (21). This hypothesis is in line with the results of our study, where breathlessness, the most common chief complaint, accounts for 65.4% of total cases, followed by cough (57,7%) and fever (42,3%). These clinical spectrums are similar to previous findings in some countries (10, 13, 22), and Indonesia (23), except for the high proportion of breathlessness symptoms. COVID-19 might share several symptoms that mimic symptoms of worsening cardiac condition in CHD, as can be seen in heart failure (24).

### Pharmacology effects on congenital heart disease with COVID-19

Concerns have been raised about the potentially harmful effects of cardiovascular drugs when used during SARS-CoV-2 infection. Beta-blockers are primarily used to treat heart failure in patients with CHD or dilated cardiomyopathy, to reduce left ventricular outflow tract obstruction in patients with hypertrophic cardiomyopathy, and to prevent ventricular tachycardia in patients with long QT syndrome. Amiodarone, Sotalol, and Flecainide can interact with other QT prolonging medications like Ritonavir/Lopinavir and Chloroquine. However, these medications are critical and should not be stopped in these extremely stressful situations where tachycardia paroxysms may occur (24). Diuretics and digoxin should be used with caution. Digoxin action is dependent on potassium/calcium levels, and toxicity may occur during COVID-19 vomiting or diarrhea episodes. Diuretics can cause electrolyte imbalance and dehydration, which can be harmful to some patients. Because there is no evidence that most of these drugs are harmful in the context of COVID-19, the general recommendation is to continue taking them unless contraindicated or side effects are observed (24).

Only the safety of Renin-angiotensin-aldosterone system (RAAS) inhibitors was called into question shortly after the pandemic began. In children with CHD and heart failure, RAAS is commonly used to reduce afterload and avoid abnormal myocardial re-modulation. It has been argued that RAAS inhibitors and angiotensin receptor blockers (ARBs) may up-regulate ACE2, thereby increasing susceptibility to the virus and consequently may cause a more severe infection. However, there was mechanistic evidence from other coronaviruses that the downregulation of ACE2 in infected patients leads to acute lung injury, therefore the upregulation of ACE2 due to RAAS inhibitors could mitigate this effect (36–41). The data is currently insufficient to make a firm recommendation to discontinue these medications in children who had previously been prescribed them to treat their cardiac condition. As a result, regardless of SARS-CoV-2 infection, the leading international cardiovascular scientific societies recommend continuing or initiating ACEI/ARB treatment in patients with heart disease when indicated (24).

## Limitation

There are no reliable data regarding the morbidity and mortality burden of CHD patients with COVID-19. Only a few studies focused on children with CHD and COVID-19, mostly limited to sporadic case reports or small case series. A limited number of subjects in this study may be due to under-screening or under-testing of the people in Indonesia, and the study population comprised only hospitalized patients.

## Conclusion

We observed a relatively mild COVID-19 clinical course despite prior research showing that patients with cardiovascular comorbidities, such as CHD, have a higher case-fatality rate. This could be because of the smaller sample size, non-standardized diagnosis, severity, treatment, and age group.

## Data availability statement

The original contributions presented in the study are included in the article/supplementary material, further inquiries can be directed to the corresponding author.

## Ethics statement

The studies involving human participants were reviewed and approved by Institutional Review Board of National Cardiovascular Center Harapan Kita No. UM.01.05/2.2.2/175/2022 has Ethically Authorized this Study. Written informed consent to participate in this study was provided by the participants' legal guardian/next of kin.

## Author contributions

SS conceived the original idea of the manuscript, and all authors discussed and agreed with the idea of the paper. SS, SO, and FP contributed in collecting data and writing the main text of the paper. BM, OL, DS, and YK performed statistical analyses and also helped in writing the paper. The manuscript was proofread and accepted by all authors.

## Conflict of interest

The authors declare that the research was conducted in the absence of any commercial or financial relationships that could be construed as a potential conflict of interest.

## Publisher's note

All claims expressed in this article are solely those of the authors and do not necessarily represent those of their affiliated organizations, or those of the publisher, the editors and the reviewers. Any product that may be evaluated in this article, or claim that may be made by its manufacturer, is not guaranteed or endorsed by the publisher.
